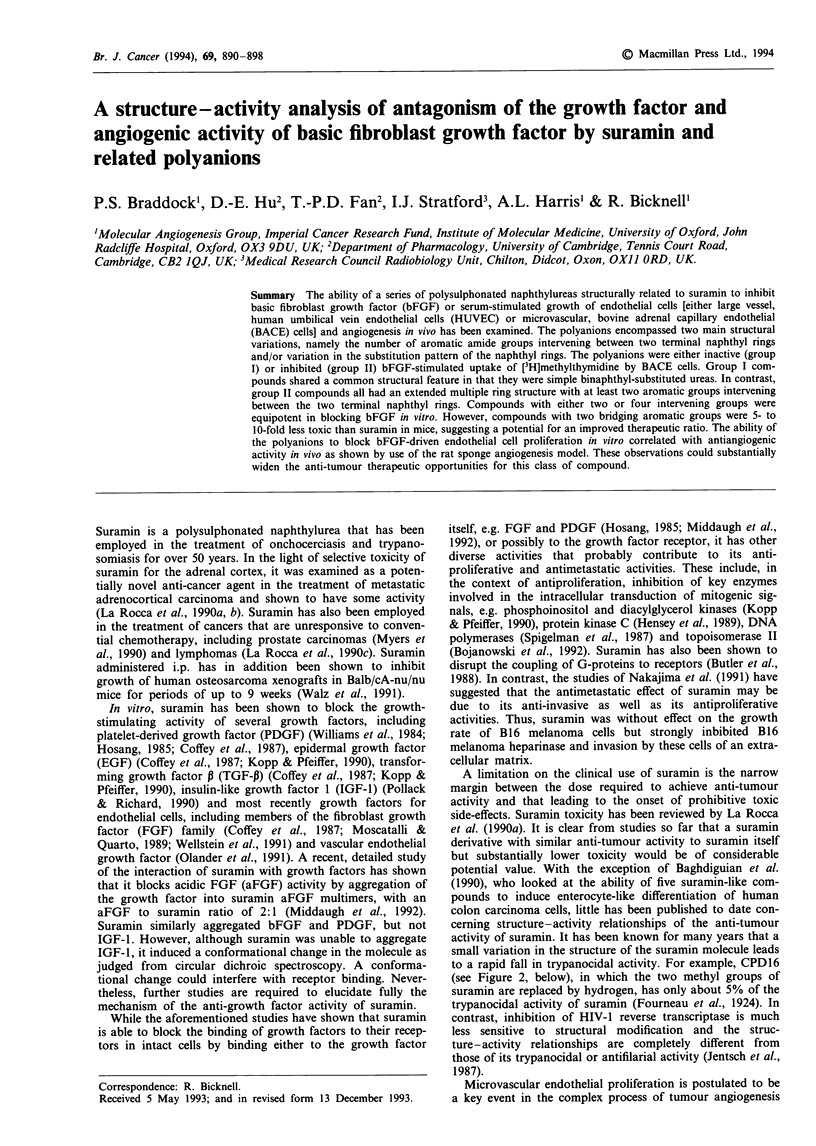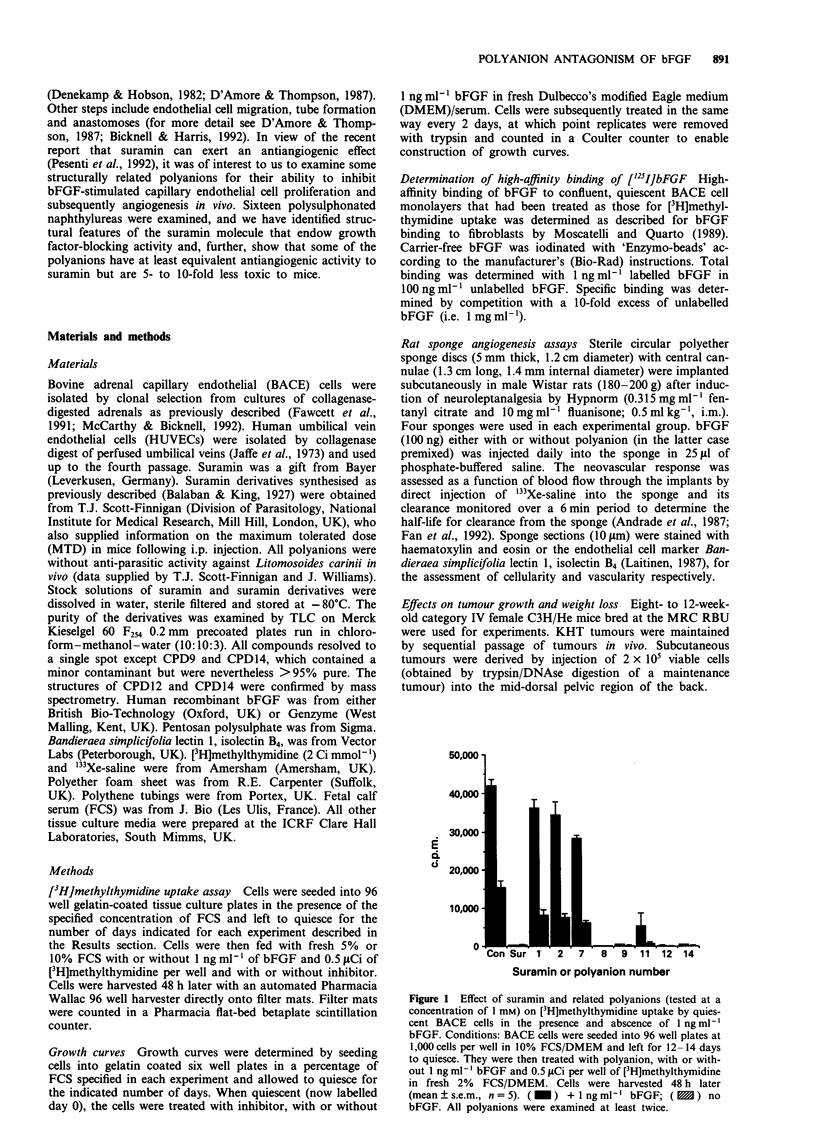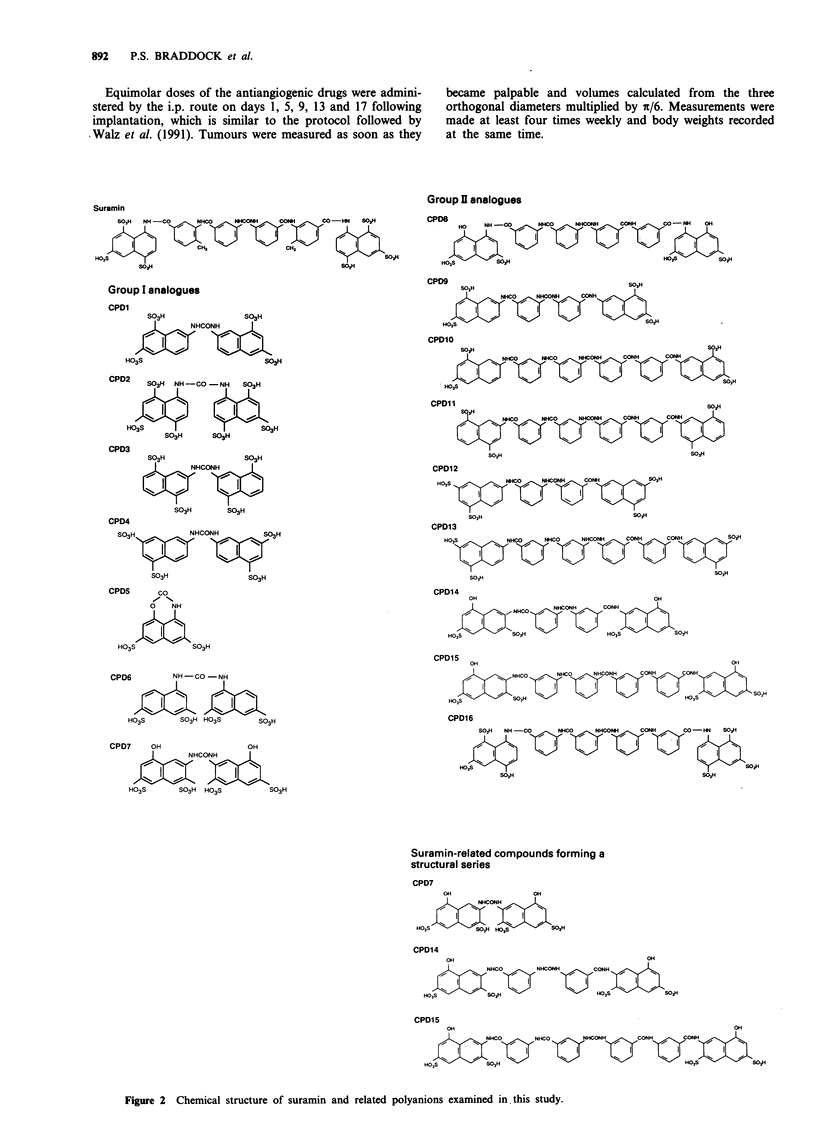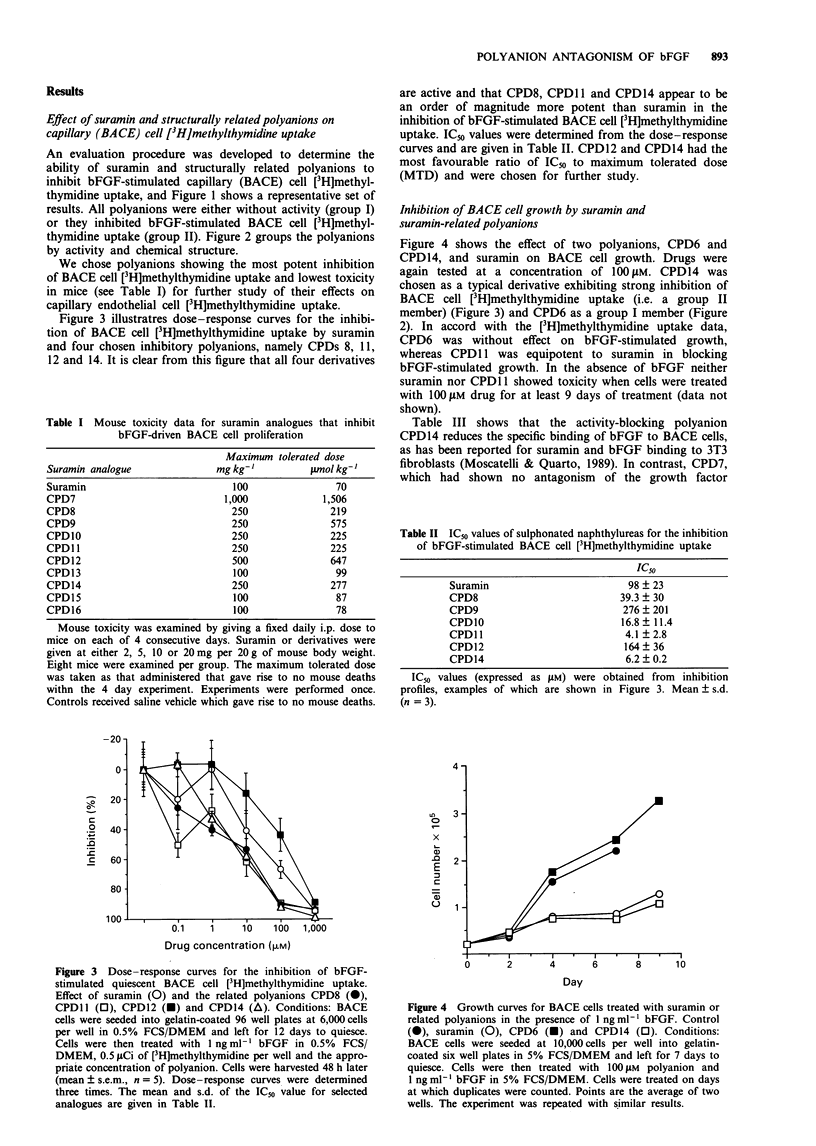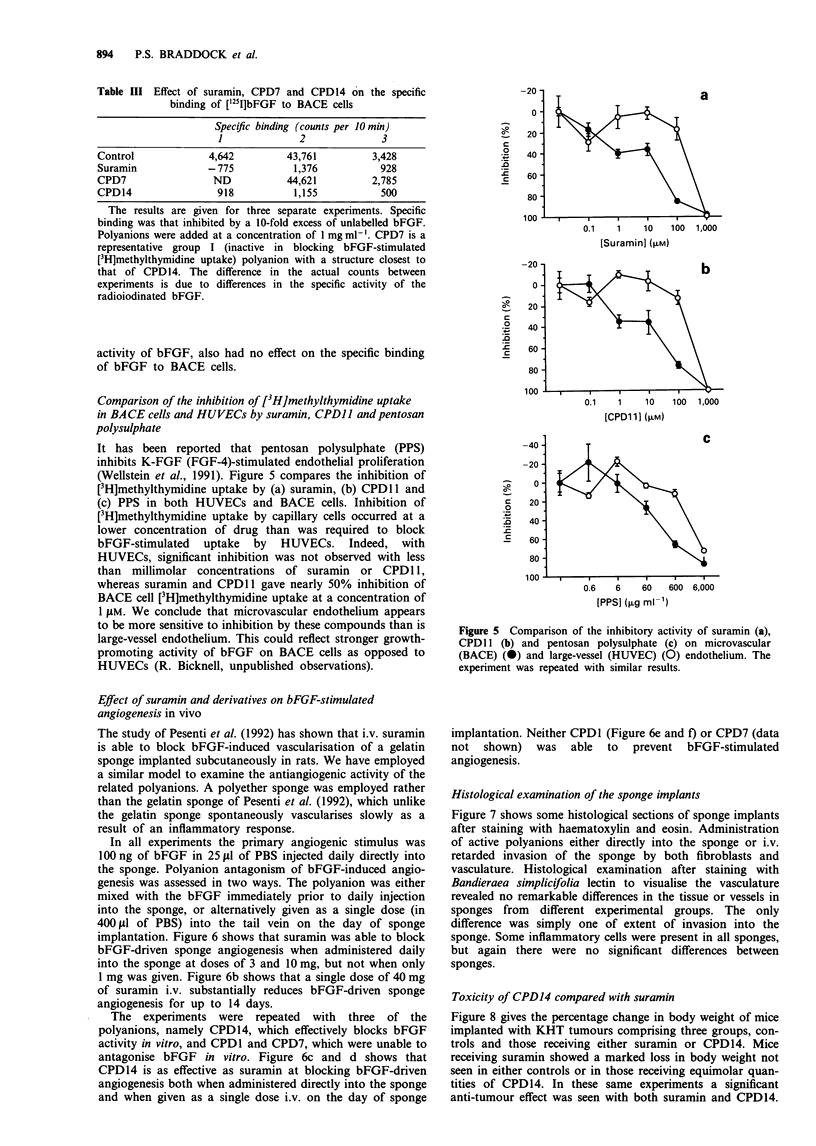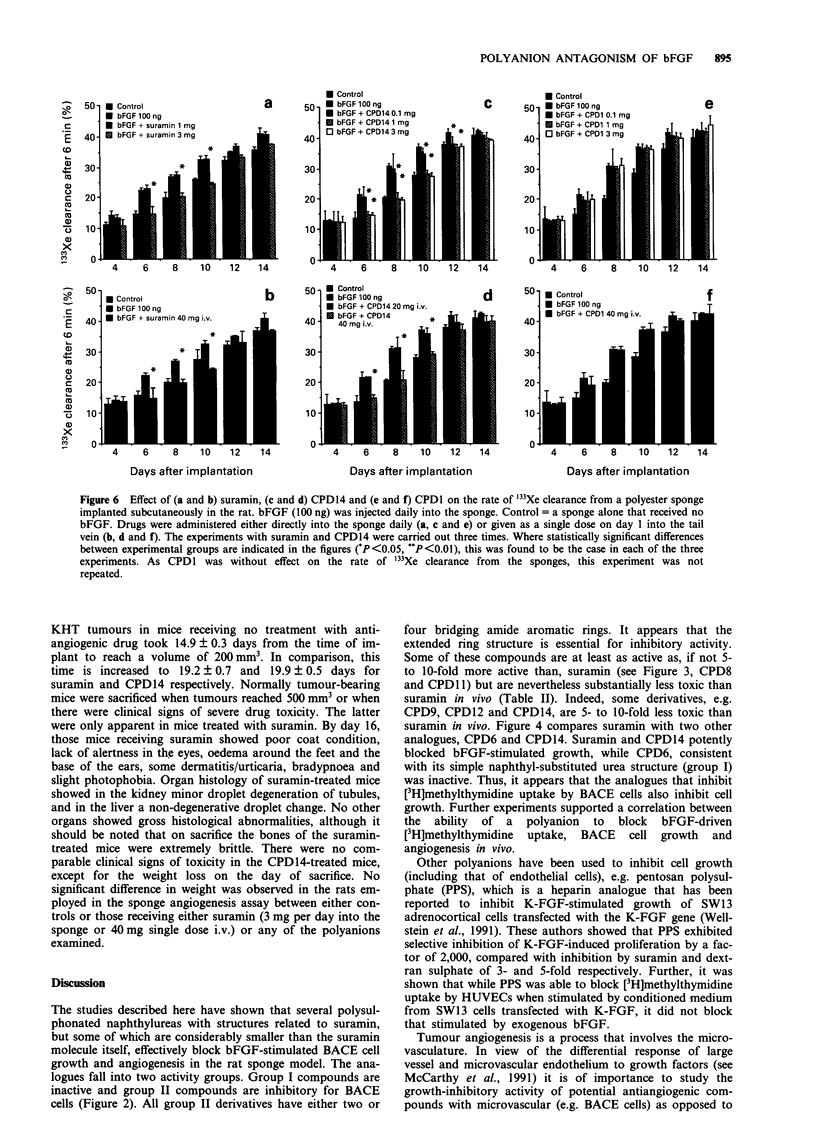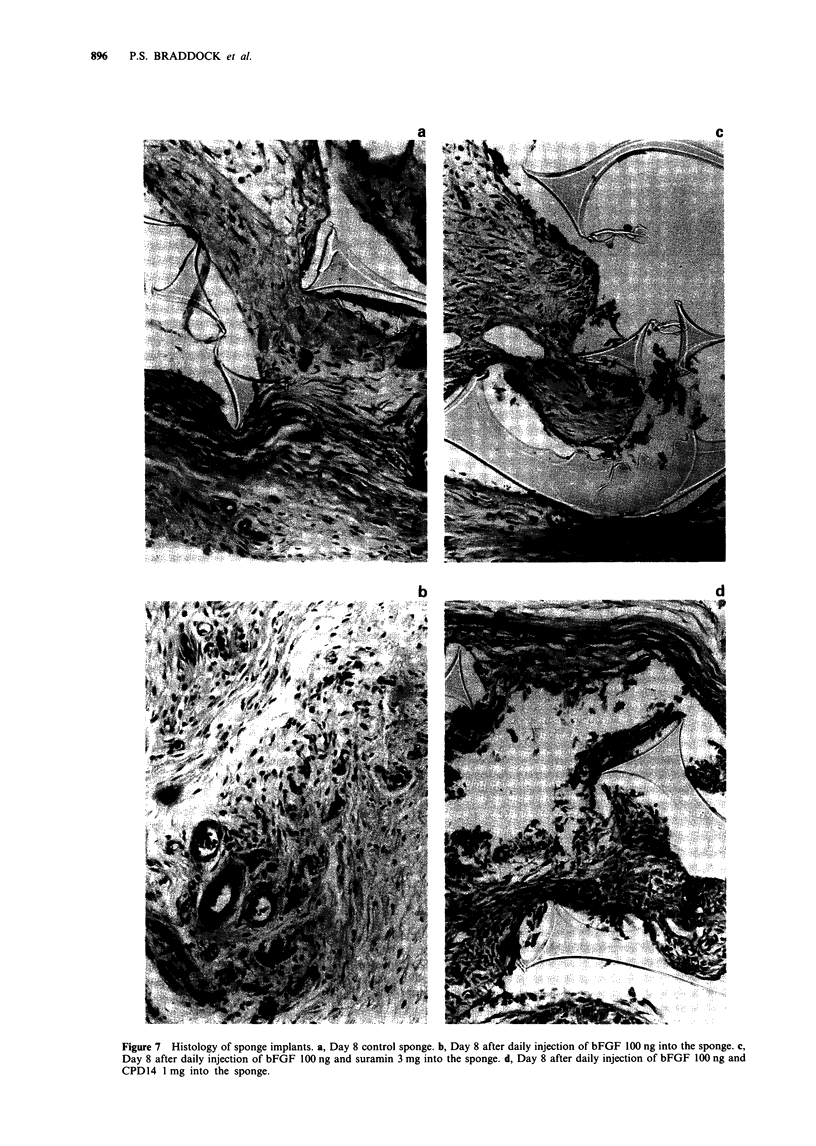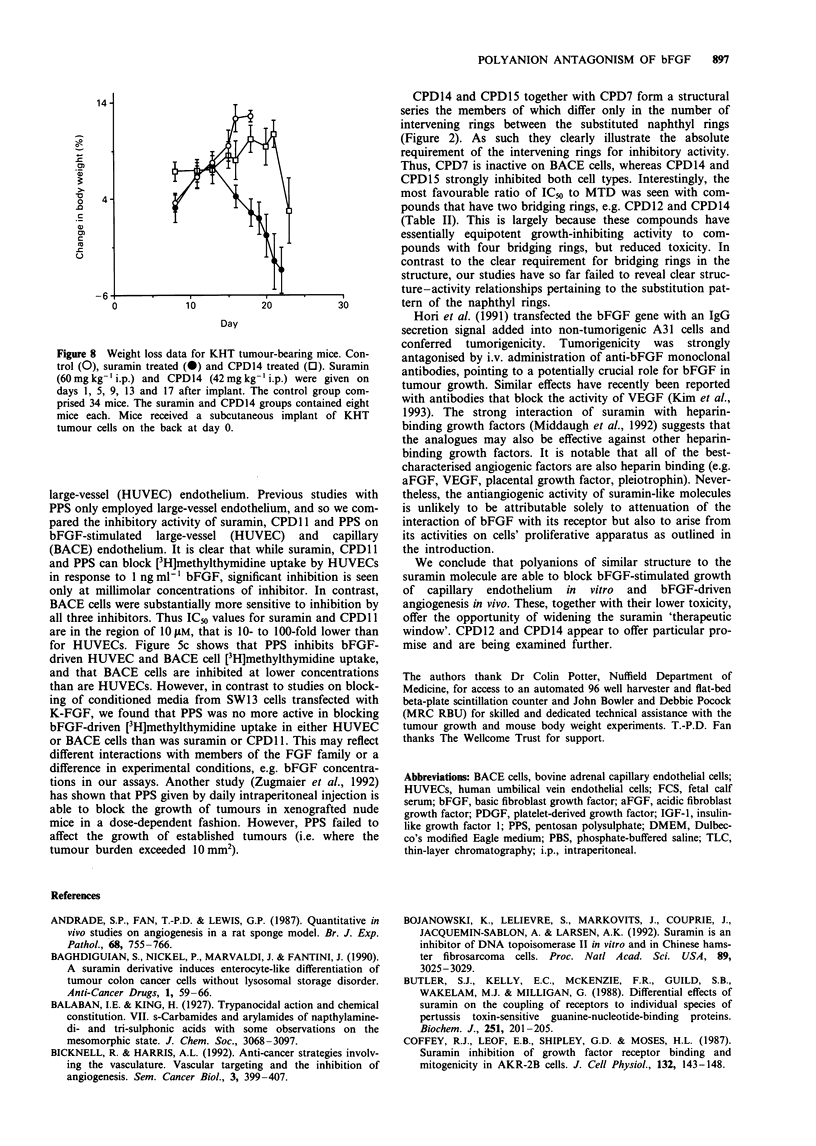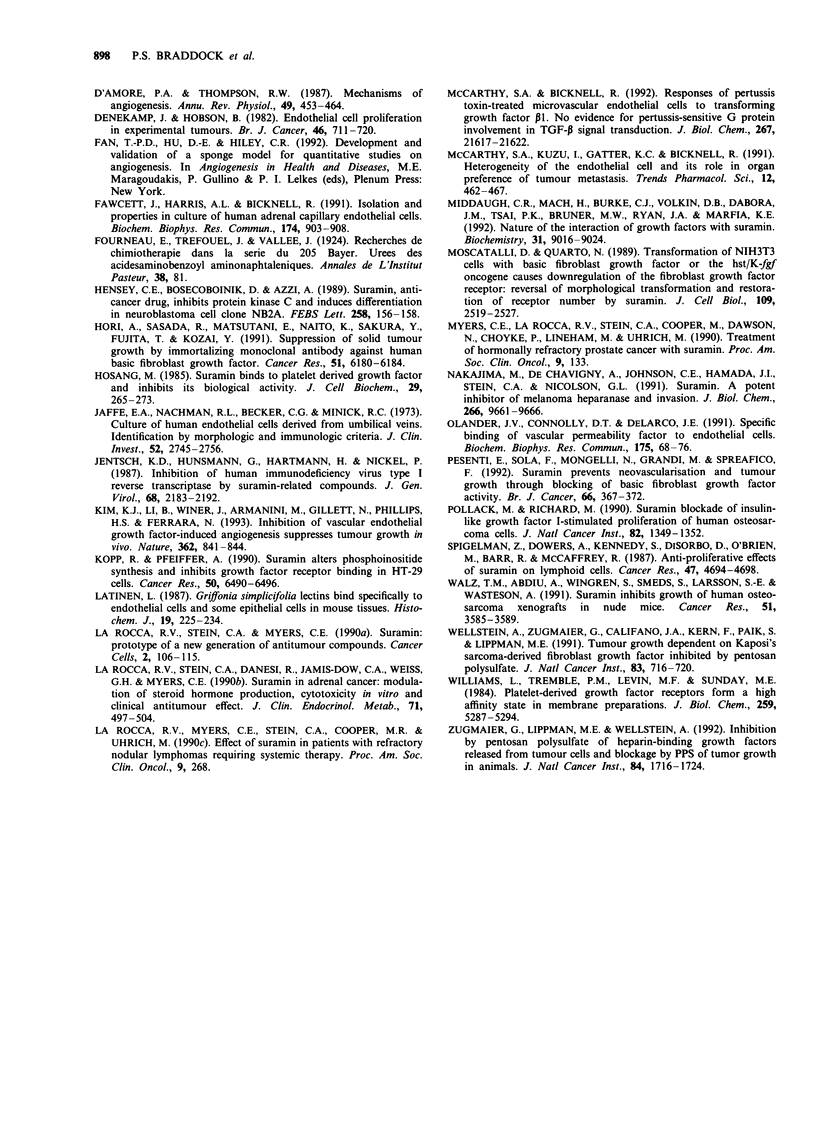# A structure-activity analysis of antagonism of the growth factor and angiogenic activity of basic fibroblast growth factor by suramin and related polyanions.

**DOI:** 10.1038/bjc.1994.172

**Published:** 1994-05

**Authors:** P. S. Braddock, D. E. Hu, T. P. Fan, I. J. Stratford, A. L. Harris, R. Bicknell

**Affiliations:** Institute of Molecular Medicine, University of Oxford, John Radcliffe Hospital, UK.

## Abstract

**Images:**


Br. J. Cancer (1994), 69, 890-898 © Macmillan Press Ltd., 1994

### A structure-activity analysis of antagonism of the growth factor and angiogenic activity of basic fibroblast growth factor by suramin and related polyanions

P.S. Braddock[1], D.-E. Hu[2], T.-P.D. Fan[2], I.J. Stratford[3], A.L. Harris[1] & R. Bicknell[1]

[1]*Molecular Angiogenesis Group, Imperial Cancer Research Fund, Institute of Molecular Medicine, University of Oxford, John Radcliffe Hospital, Oxford, OX3 9DU, UK;* [2]*Department of Pharmacology, University of Cambridge, Tennis Court Road, Cambridge, CB2 1QJ, UK;* [3]*Medical Research Council Radiobiology Unit, Chilton, Didcot, Oxon, OX11 0RD, UK.*

**Summary** The ability of a series of polysulphonated naphthylureas structurally related to suramin to inhibit basic fibroblast growth factor (bFGF) or serum-stimulated growth of endothelial cells [either large vessel, human umbilical vein endothelial cells (HUVEC) or microvascular, bovine adrenal capillary endothelial (BACE) cells] and angiogenesis *in vivo* has been examined. The polyanions encompassed two main structural variations, namely the number of aromatic amide groups intervening between two terminal naphthyl rings and/or variation in the substitution pattern of the naphthyl rings. The polyanions were either inactive (group I) or inhibited (group II) bFGF-stimulated uptake of [$^3$H]methylthymidine by BACE cells. Group I compounds shared a common structural feature in that they were simple binaphthyl-substituted ureas. In contrast, group II compounds all had an extended multiple ring structure with at least two aromatic groups intervening between the two terminal naphthyl rings. Compounds with either two or four intervening groups were equipotent in blocking bFGF *in vitro*. However, compounds with two bridging aromatic groups were 5- to 10-fold less toxic than suramin in mice, suggesting a potential for an improved therapeutic ratio. The ability of the polyanions to block bFGF-driven endothelial cell proliferation *in vitro* correlated with antiangiogenic activity *in vivo* as shown by use of the rat sponge angiogenesis model. These observations could substantially widen the anti-tumour therapeutic opportunities for this class of compound.

Suramin is a polysulphonated naphthylurea that has been employed in the treatment of onchocerciasis and trypanosomiasis for over 50 years. In the light of selective toxicity of suramin for the adrenal cortex, it was examined as a potentially novel anti-cancer agent in the treatment of metastatic adrenocortical carcinoma and shown to have some activity (La Rocca *et al.*, 1990*a*, *b*). Suramin has also been employed in the treatment of cancers that are unresponsive to conventional chemotherapy, including prostate carcinomas (Myers *et al.*, 1990) and lymphomas (La Rocca *et al.*, 1990*c*). Suramin administered i.p. has in addition been shown to inhibit growth of human osteosarcoma xenografts in Balb/cA-nu/nu mice for periods of up to 9 weeks (Walz *et al.*, 1991).

*In vitro*, suramin has been shown to block the growth-stimulating activity of several growth factors, including platelet-derived growth factor (PDGF) (Williams *et al.*, 1984; Hosang, 1985; Coffey *et al.*, 1987), epidermal growth factor (EGF) (Coffey *et al.*, 1987; Kopp & Pfeiffer, 1990), transforming growth factor $\beta$ (TGF-$\beta$) (Coffey *et al.*, 1987; Kopp & Pfeiffer, 1990), insulin-like growth factor 1 (IGF-1) (Pollack & Richard, 1990) and most recently growth factors for endothelial cells, including members of the fibroblast growth factor (FGF) family (Coffey *et al.*, 1987; Moscatalli & Quarto, 1989; Wellstein *et al.*, 1991) and vascular endothelial growth factor (Olander *et al.*, 1991). A recent, detailed study of the interaction of suramin with growth factors has shown that it blocks acidic FGF (aFGF) activity by aggregation of the growth factor into suramin aFGF multimers, with an aFGF to suramin ratio of 2:1 (Middaugh *et al.*, 1992). Suramin similarly aggregated bFGF and PDGF, but not IGF-1. However, although suramin was unable to aggregate IGF-1, it induced a conformational change in the molecule as judged from circular dichroic spectroscopy. A conformational change could interfere with receptor binding. Nevertheless, further studies are required to elucidate fully the mechanism of the anti-growth factor activity of suramin.

While the aforementioned studies have shown that suramin is able to block the binding of growth factors to their receptors in intact cells by binding either to the growth factor itself, e.g. FGF and PDGF (Hosang, 1985; Middaugh *et al.*, 1992), or possibly to the growth factor receptor, it has other diverse activities that probably contribute to its antiproliferative and antimetastatic activities. These include, in the context of antiproliferation, inhibition of key enzymes involved in the intracellular transduction of mitogenic signals, e.g. phosphoinositol and diacylglycerol kinases (Kopp & Pfeiffer, 1990), protein kinase C (Hensey *et al.*, 1989), DNA polymerases (Spigelman *et al.*, 1987) and topoisomerase II (Bojanowski *et al.*, 1992). Suramin has also been shown to disrupt the coupling of G-proteins to receptors (Butler *et al.*, 1988). In contrast, the studies of Nakajima *et al.* (1991) have suggested that the antimetastatic effect of suramin may be due to its anti-invasive as well as its antiproliferative activities. Thus, suramin was without effect on the growth rate of B16 melanoma cells but strongly inbibited B16 melanoma heparinase and invasion by these cells of an extracellular matrix.

A limitation on the clinical use of suramin is the narrow margin between the dose required to achieve anti-tumour activity and that leading to the onset of prohibitive toxic side-effects. Suramin toxicity has been reviewed by La Rocca *et al.* (1990*a*). It is clear from studies so far that a suramin derivative with similar anti-tumour activity to suramin itself but substantially lower toxicity would be of considerable potential value. With the exception of Baghdiguian *et al.* (1990), who looked at the ability of five suramin-like compounds to induce enterocyte-like differentiation of human colon carcinoma cells, little has been published to date concerning structure-activity relationships of the anti-tumour activity of suramin. It has been known for many years that a small variation in the structure of the suramin molecule leads to a rapid fall in trypanocidal activity. For example, CPD16 (see Figure 2, below), in which the two methyl groups of suramin are replaced by hydrogen, has only about 5% of the trypanocidal activity of suramin (Fourneau *et al.*, 1924). In contrast, inhibition of HIV-1 reverse transcriptase is much less sensitive to structural modification and the structure-activity relationships are completely different from those of its trypanocidal or antifilarial activity (Jentsch *et al.*, 1987).

Microvascular endothelial proliferation is postulated to be a key event in the complex process of tumour angiogenesis

Correspondence: R. Bicknell.
Received 5 May 1993; and in revised form 13 December 1993.

(Denekamp & Hobson, 1982; D'Amore & Thompson, 1987). Other steps include endothelial cell migration, tube formation and anastomoses (for more detail see D'Amore & Thompson, 1987; Bicknell & Harris, 1992). In view of the recent report that suramin can exert an antiangiogenic effect (Pesenti *et al.*, 1992), it was of interest to us to examine some structurally related polyanions for their ability to inhibit bFGF-stimulated capillary endothelial cell proliferation and subsequently angiogenesis *in vivo*. Sixteen polysulphonated naphthylureas were examined, and we have identified structural features of the suramin molecule that endow growth factor-blocking activity and, further, show that some of the polyanions have at least equivalent antiangiogenic activity to suramin but are 5- to 10-fold less toxic to mice.

### Materials and methods

### *Materials*

Bovine adrenal capillary endothelial (BACE) cells were isolated by clonal selection from cultures of collagenase-digested adrenals as previously described (Fawcett *et al.*, 1991; McCarthy & Bicknell, 1992). Human umbilical vein endothelial cells (HUVECs) were isolated by collagenase digest of perfused umbilical veins (Jaffe *et al.*, 1973) and used up to the fourth passage. Suramin was a gift from Bayer (Leverkusen, Germany). Suramin derivatives synthesised as previously described (Balaban & King, 1927) were obtained from T.J. Scott-Finnigan (Division of Parasitology, National Institute for Medical Research, Mill Hill, London, UK), who also supplied information on the maximum tolerated dose (MTD) in mice following i.p. injection. All polyanions were without anti-parasitic activity against *Litomosoides carinii in vivo* (data supplied by T.J. Scott-Finnigan and J. Williams). Stock solutions of suramin and suramin derivatives were dissolved in water, sterile filtered and stored at $-80°C$. The purity of the derivatives was examined by TLC on Merck Kieselgel 60 $F_{254}$ 0.2 mm precoated plates run in chloroform–methanol–water (10:10:3). All compounds resolved to a single spot except CPD9 and CPD14, which contained a minor contaminant but were nevertheless >95% pure. The structures of CPD12 and CPD14 were confirmed by mass spectrometry. Human recombinant bFGF was from either British Bio-Technology (Oxford, UK) or Genzyme (West Malling, Kent, UK). Pentosan polysulphate was from Sigma. *Bandieraea simplicifolia* lectin 1, isolectin $B_4$, was from Vector Labs (Peterborough, UK). [$^3$H]methylthymidine (2 Ci mmol$^{-1}$) and $^{133}$Xe-saline were from Amersham (Amersham, UK). Polyether foam sheet was from R.E. Carpenter (Suffolk, UK). Polythene tubings were from Portex, UK. Fetal calf serum (FCS) was from J. Bio (Les Ulis, France). All other tissue culture media were prepared at the ICRF Clare Hall Laboratories, South Mimms, UK.

### *Methods*

*[$^3$H]methylthymidine uptake assay* Cells were seeded into 96 well gelatin-coated tissue culture plates in the presence of the specified concentration of FCS and left to quiesce for the number of days indicated for each experiment described in the Results section. Cells were then fed with fresh 5% or 10% FCS with or without 1 ng ml$^{-1}$ of bFGF and 0.5 $\mu$Ci of [$^3$H]methylthymidine per well and with or without inhibitor. Cells were harvested 48 h later with an automated Pharmacia Wallac 96 well harvester directly onto filter mats. Filter mats were counted in a Pharmacia flat-bed betaplate scintillation counter.

*Growth curves* Growth curves were determined by seeding cells into gelatin coated six well plates in a percentage of FCS specified in each experiment and allowed to quiesce for the indicated number of days. When quiescent (now labelled day 0), the cells were treated with inhibitor, with or without 1 ng ml$^{-1}$ bFGF in fresh Dulbecco's modified Eagle medium (DMEM)/serum. Cells were subsequently treated in the same way every 2 days, at which point replicates were removed with trypsin and counted in a Coulter counter to enable construction of growth curves.

*Determination of high-affinity binding of [$^{125}$I]bFGF* High-affinity binding of bFGF to confluent, quiescent BACE cell monolayers that had been treated as those for [$^3$H]methylthymidine uptake was determined as described for bFGF binding to fibroblasts by Moscatelli and Quarto (1989). Carrier-free bFGF was iodinated with 'Enzymo-beads' according to the manufacturer's (Bio-Rad) instructions. Total binding was determined with 1 ng ml$^{-1}$ labelled bFGF in 100 ng ml$^{-1}$ unlabelled bFGF. Specific binding was determined by competition with a 10-fold excess of unlabelled bFGF (i.e. 1 mg ml$^{-1}$).

*Rat sponge angiogenesis assays* Sterile circular polyether sponge discs (5 mm thick, 1.2 cm diameter) with central cannulae (1.3 cm long, 1.4 mm internal diameter) were implanted subcutaneously in male Wistar rats (180–200 g) after induction of neuroleptanalgesia by Hypnorm (0.315 mg ml$^{-1}$ fentanyl citrate and 10 mg ml$^{-1}$ fluanisone; 0.5 ml kg$^{-1}$, i.m.). Four sponges were used in each experimental group. bFGF (100 ng) either with or without polyanion (in the latter case premixed) was injected daily into the sponge in 25 $\mu$l of phosphate-buffered saline. The neovascular response was assessed as a function of blood flow through the implants by direct injection of $^{133}$Xe-saline into the sponge and its clearance monitored over a 6 min period to determine the half-life for clearance from the sponge (Andrade *et al.*, 1987; Fan *et al.*, 1992). Sponge sections (10 $\mu$m) were stained with haematoxylin and eosin or the endothelial cell marker *Bandieraea simplicifolia* lectin 1, isolectin $B_4$ (Laitinen, 1987), for the assessment of cellularity and vascularity respectively.

*Effects on tumour growth and weight loss* Eight- to 12-week-old category IV female C3H/He mice bred at the MRC RBU were used for experiments. KHT tumours were maintained by sequential passage of tumours *in vivo*. Subcutaneous tumours were derived by injection of $2 \times 10^5$ viable cells (obtained by trypsin/DNAse digestion of a maintenance tumour) into the mid-dorsal pelvic region of the back.

**Figure 1** Effect of suramin and related polyanions (tested at a concentration of 1 mM) on [$^3$H]methylthymidine uptake by quiescent BACE cells in the presence and abscence of 1 ng ml$^{-1}$ bFGF. Conditions: BACE cells were seeded into 96 well plates at 1,000 cells per well in 10% FCS/DMEM and left for 12–14 days to quiesce. They were then treated with polyanion, with or without 1 ng ml$^{-1}$ bFGF and 0.5 $\mu$Ci per well of [$^3$H]methylthymidine in fresh 2% FCS/DMEM. Cells were harvested 48 h later (mean ± s.e.m., $n = 5$). (■) + 1 ng ml$^{-1}$ bFGF; (▨) no bFGF. All polyanions were examined at least twice.

Equimolar doses of the antiangiogenic drugs were administered by the i.p. route on days 1, 5, 9, 13 and 17 following implantation, which is similar to the protocol followed by Walz *et al.* (1991). Tumours were measured as soon as they became palpable and volumes calculated from the three orthogonal diameters multiplied by $\pi/6$. Measurements were made at least four times weekly and body weights recorded at the same time.

**Figure 2** Chemical structure of suramin and related polyanions examined in this study.

### Results

### *Effect of suramin and structurally related polyanions on capillary (BACE) cell [$^3$H]methylthymidine uptake*

An evaluation procedure was developed to determine the ability of suramin and structurally related polyanions to inhibit bFGF-stimulated capillary (BACE) cell [$^3$H]methylthymidine uptake, and Figure 1 shows a representative set of results. All polyanions were either without activity (group I) or they inhibited bFGF-stimulated BACE cell [$^3$H]methylthymidine uptake (group II). Figure 2 groups the polyanions by activity and chemical structure.

We chose polyanions showing the most potent inhibition of BACE cell [$^3$H]methylthymidine uptake and lowest toxicity in mice (see Table I) for further study of their effects on capillary endothelial cell [$^3$H]methylthymidine uptake.

Figure 3 illustratres dose-response curves for the inhibition of BACE cell [$^3$H]methylthymidine uptake by suramin and four chosen inhibitory polyanions, namely CPDs 8, 11, 12 and 14. It is clear from this figure that all four derivatives are active and that CPD8, CPD11 and CPD14 appear to be an order of magnitude more potent than suramin in the inhibition of bFGF-stimulated BACE cell [$^3$H]methylthymidine uptake. $IC_{50}$ values were determined from the dose-response curves and are given in Table II. CPD12 and CPD14 had the most favourable ratio of $IC_{50}$ to maximum tolerated dose (MTD) and were chosen for further study.

**Table I** Mouse toxicity data for suramin analogues that inhibit bFGF-driven BACE cell proliferation

| *Suramin analogue* | *Maximum tolerated dose* *mg kg$^{-1}$* | *µmol kg$^{-1}$* |
|---|---|---|
| Suramin | 100 | 70 |
| CPD7 | 1,000 | 1,506 |
| CPD8 | 250 | 219 |
| CPD9 | 250 | 575 |
| CPD10 | 250 | 225 |
| CPD11 | 250 | 225 |
| CPD12 | 500 | 647 |
| CPD13 | 100 | 99 |
| CPD14 | 250 | 277 |
| CPD15 | 100 | 87 |
| CPD16 | 100 | 78 |

Mouse toxicity was examined by giving a fixed daily i.p. dose to mice on each of 4 consecutive days. Suramin or derivatives were given at either 2, 5, 10 or 20 mg per 20 g of mouse body weight. Eight mice were examined per group. The maximum tolerated dose was taken as that administered that gave rise to no mouse deaths withn the 4 day experiment. Experiments were performed once. Controls received saline vehicle which gave rise to no mouse deaths.

**Figure 3** Dose-response curves for the inhibition of bFGF-stimulated quiescent BACE cell [$^3$H]methylthymidine uptake. Effect of suramin (○) and the related polyanions CPD8 (●), CPD11 (□), CPD12 (■) and CPD14 (△). Conditions: BACE cells were seeded into gelatin-coated 96 well plates at 6,000 cells per well in 0.5% FCS/DMEM and left for 12 days to quiesce. Cells were then treated with 1 ng ml$^{-1}$ bFGF in 0.5% FCS/DMEM, 0.5 µCi of [$^3$H]methylthymidine per well and the appropriate concentration of polyanion. Cells were harvested 48 h later (mean ± s.e.m., $n = 5$). Dose-response curves were determined three times. The mean and s.d. of the $IC_{50}$ value for selected analogues are given in Table II.

### *Inhibition of BACE cell growth by suramin and suramin-related polyanions*

Figure 4 shows the effect of two polyanions, CPD6 and CPD14, and suramin on BACE cell growth. Drugs were again tested at a concentration of 100 µM. CPD14 was chosen as a typical derivative exhibiting strong inhibition of BACE cell [$^3$H]methylthymidine uptake (i.e. a group II member) (Figure 3) and CPD6 as a group I member (Figure 2). In accord with the [$^3$H]methylthymidine uptake data, CPD6 was without effect on bFGF-stimulated growth, whereas CPD11 was equipotent to suramin in blocking bFGF-stimulated growth. In the absence of bFGF neither suramin nor CPD11 showed toxicity when cells were treated with 100 µM drug for at least 9 days of treatment (data not shown).

Table III shows that the activity-blocking polyanion CPD14 reduces the specific binding of bFGF to BACE cells, as has been reported for suramin and bFGF binding to 3T3 fibroblasts (Moscatelli & Quarto, 1989). In contrast, CPD7, which had shown no antagonism of the growth factor

**Table II** $IC_{50}$ values of sulphonated naphthylureas for the inhibition of bFGF-stimulated BACE cell [$^3$H]methylthymidine uptake

| | *$IC_{50}$* |
|---|---|
| Suramin | 98 ± 23 |
| CPD8 | 39.3 ± 30 |
| CPD9 | 276 ± 201 |
| CPD10 | 16.8 ± 11.4 |
| CPD11 | 4.1 ± 2.8 |
| CPD12 | 164 ± 36 |
| CPD14 | 6.2 ± 0.2 |

$IC_{50}$ values (expressed as µM) were obtained from inhibition profiles, examples of which are shown in Figure 3. Mean ± s.d. ($n = 3$).

**Figure 4** Growth curves for BACE cells treated with suramin or related polyanions in the presence of 1 ng ml$^{-1}$ bFGF. Control (●), suramin (○), CPD6 (■) and CPD14 (□). Conditions: BACE cells were seeded at 10,000 cells per well into gelatin-coated six well plates in 5% FCS/DMEM and left for 7 days to quiesce. Cells were then treated with 100 µM polyanion and 1 ng ml$^{-1}$ bFGF in 5% FCS/DMEM. Cells were treated on days at which duplicates were counted. Points are the average of two wells. The experiment was repeated with similar results.

**Table III** Effect of suramin, CPD7 and CPD14 on the specific binding of [$^{125}$I]bFGF to BACE cells

| | Specific binding (counts per 10 min) 1 | 2 | 3 |
|---|---|---|---|
| Control | 4,642 | 43,761 | 3,428 |
| Suramin | –775 | 1,376 | 928 |
| CPD7 | ND | 44,621 | 2,785 |
| CPD14 | 918 | 1,155 | 500 |

The results are given for three separate experiments. Specific binding was that inhibited by a 10-fold excess of unlabelled bFGF. Polyanions were added at a concentration of 1 mg ml$^{-1}$. CPD7 is a representative group I (inactive in blocking bFGF-stimulated [$^3$H]methylthymidine uptake) polyanion with a structure closest to that of CPD14. The difference in the actual counts between experiments is due to differences in the specific activity of the radioiodinated bFGF.

activity of bFGF, also had no effect on the specific binding of bFGF to BACE cells.

*Comparison of the inhibition of [$^3$H]methylthymidine uptake in BACE cells and HUVECs by suramin, CPD11 and pentosan polysulphate*

It has been reported that pentosan polysulphate (PPS) inhibits K-FGF (FGF-4)-stimulated endothelial proliferation (Wellstein *et al.*, 1991). Figure 5 compares the inhibition of [$^3$H]methylthymidine uptake by (a) suramin, (b) CPD11 and (c) PPS in both HUVECs and BACE cells. Inhibition of [$^3$H]methylthymidine uptake by capillary cells occurred at a lower concentration of drug than was required to block bFGF-stimulated uptake by HUVECs. Indeed, with HUVECs, significant inhibition was not observed with less than millimolar concentrations of suramin or CPD11, whereas suramin and CPD11 gave nearly 50% inhibition of BACE cell [$^3$H]methylthymidine uptake at a concentration of 1 µM. We conclude that microvascular endothelium appears to be more sensitive to inhibition by these compounds than is large-vessel endothelium. This could reflect stronger growth-promoting activity of bFGF on BACE cells as opposed to HUVECs (R. Bicknell, unpublished observations).

**Figure 5** Comparison of the inhibitory activity of suramin (**a**), CPD11 (**b**) and pentosan polysulphate (**c**) on microvascular (BACE) (●) and large-vessel (HUVEC) (○) endothelium. The experiment was repeated with similar results.

*Effect of suramin and derivatives on bFGF-stimulated angiogenesis* in vivo

The study of Pesenti *et al.* (1992) has shown that i.v. suramin is able to block bFGF-induced vascularisation of a gelatin sponge implanted subcutaneously in rats. We have employed a similar model to examine the antiangiogenic activity of the related polyanions. A polyether sponge was employed rather than the gelatin sponge of Pesenti *et al.* (1992), which unlike the gelatin sponge spontaneously vascularises slowly as a result of an inflammatory response.

In all experiments the primary angiogenic stimulus was 100 ng of bFGF in 25 µl of PBS injected daily directly into the sponge. Polyanion antagonism of bFGF-induced angiogenesis was assessed in two ways. The polyanion was either mixed with the bFGF immediately prior to daily injection into the sponge, or alternatively given as a single dose (in 400 µl of PBS) into the tail vein on the day of sponge implantation. Figure 6 shows that suramin was able to block bFGF-driven sponge angiogenesis when administered daily into the sponge at doses of 3 and 10 mg, but not when only 1 mg was given. Figure 6b shows that a single dose of 40 mg of suramin i.v. substantially reduces bFGF-driven sponge angiogenesis for up to 14 days.

The experiments were repeated with three of the polyanions, namely CPD14, which effectively blocks bFGF activity *in vitro*, and CPD1 and CPD7, which were unable to antagonise bFGF *in vitro*. Figure 6c and d shows that CPD14 is as effective as suramin at blocking bFGF-driven angiogenesis both when administered directly into the sponge and when given as a single dose i.v. on the day of sponge implantation. Neither CPD1 (Figure 6e and f) or CPD7 (data not shown) was able to prevent bFGF-stimulated angiogenesis.

*Histological examination of the sponge implants*

Figure 7 shows some histological sections of sponge implants after staining with haematoxylin and eosin. Administration of active polyanions either directly into the sponge or i.v. retarded invasion of the sponge by both fibroblasts and vasculature. Histological examination after staining with *Bandieraea simplicifolia* lectin to visualise the vasculature revealed no remarkable differences in the tissue or vessels in sponges from different experimental groups. The only difference was simply one of extent of invasion into the sponge. Some inflammatory cells were present in all sponges, but again there were no significant differences between sponges.

*Toxicity of CPD14 compared with suramin*

Figure 8 gives the percentage change in body weight of mice implanted with KHT tumours comprising three groups, controls and those receiving either suramin or CPD14. Mice receiving suramin showed a marked loss in body weight not seen in either controls or in those receiving equimolar quantities of CPD14. In these same experiments a significant anti-tumour effect was seen with both suramin and CPD14.

**Figure 6** Effect of (**a** and **b**) suramin, (**c** and **d**) CPD14 and (**e** and **f**) CPD1 on the rate of $^{133}$Xe clearance from a polyester sponge implanted subcutaneously in the rat. bFGF (100 ng) was injected daily into the sponge. Control = a sponge alone that received no bFGF. Drugs were administered either directly into the sponge daily (**a**, **c** and **e**) or given as a single dose on day 1 into the tail vein (**b**, **d** and **f**). The experiments with suramin and CPD14 were carried out three times. Where statistically significant differences between experimental groups are indicated in the figures ($^{*}P<0.05$, $^{**}P<0.01$), this was found to be the case in each of the three experiments. As CPD1 was without effect on the rate of $^{133}$Xe clearance from the sponges, this experiment was not repeated.

KHT tumours in mice receiving no treatment with anti-angiogenic drug took 14.9 ± 0.3 days from the time of implant to reach a volume of 200 mm$^3$. In comparison, this time is increased to 19.2 ± 0.7 and 19.9 ± 0.5 days for suramin and CPD14 respectively. Normally tumour-bearing mice were sacrificed when tumours reached 500 mm$^3$ or when there were clinical signs of severe drug toxicity. The latter were only apparent in mice treated with suramin. By day 16, those mice receiving suramin showed poor coat condition, lack of alertness in the eyes, oedema around the feet and the base of the ears, some dermatitis/urticaria, bradypnoea and slight photophobia. Organ histology of suramin-treated mice showed in the kidney minor droplet degeneration of tubules, and in the liver a non-degenerative droplet change. No other organs showed gross histological abnormalities, although it should be noted that on sacrifice the bones of the suramin-treated mice were extremely brittle. There were no comparable clinical signs of toxicity in the CPD14-treated mice, except for the weight loss on the day of sacrifice. No significant difference in weight was observed in the rats employed in the sponge angiogenesis assay between either controls or those receiving either suramin (3 mg per day into the sponge or 40 mg single dose i.v.) or any of the polyanions examined.

### Discussion

The studies described here have shown that several polysulphonated naphthylureas with structures related to suramin, but some of which are considerably smaller than the suramin molecule itself, effectively block bFGF-stimulated BACE cell growth and angiogenesis in the rat sponge model. The analogues fall into two activity groups. Group I compounds are inactive and group II compounds are inhibitory for BACE cells (Figure 2). All group II derivatives have either two or four bridging amide aromatic rings. It appears that the extended ring structure is essential for inhibitory activity. Some of these compounds are at least as active as, if not 5- to 10-fold more active than, suramin (see Figure 3, CPD8 and CPD11) but are nevertheless substantially less toxic than suramin *in vivo* (Table II). Indeed, some derivatives, e.g. CPD9, CPD12 and CPD14, are 5- to 10-fold less toxic than suramin *in vivo*. Figure 4 compares suramin with two other analogues, CPD6 and CPD14. Suramin and CPD14 potently blocked bFGF-stimulated growth, while CPD6, consistent with its simple naphthyl-substituted urea structure (group I) was inactive. Thus, it appears that the analogues that inhibit [$^3$H]methylthymidine uptake by BACE cells also inhibit cell growth. Further experiments supported a correlation between the ability of a polyanion to block bFGF-driven [$^3$H]methylthymidine uptake, BACE cell growth and angiogenesis *in vivo*.

Other polyanions have been used to inhibit cell growth (including that of endothelial cells), e.g. pentosan polysulphate (PPS), which is a heparin analogue that has been reported to inhibit K-FGF-stimulated growth of SW13 adrenocortical cells transfected with the K-FGF gene (Wellstein *et al.*, 1991). These authors showed that PPS exhibited selective inhibition of K-FGF-induced proliferation by a factor of 2,000, compared with inhibition by suramin and dextran sulphate of 3- and 5-fold respectively. Further, it was shown that while PPS was able to block [$^3$H]methylthymidine uptake by HUVECs when stimulated by conditioned medium from SW13 cells transfected with K-FGF, it did not block that stimulated by exogenous bFGF.

Tumour angiogenesis is a process that involves the microvasculature. In view of the differential response of large vessel and microvascular endothelium to growth factors (see McCarthy *et al.*, 1991) it is of importance to study the growth-inhibitory activity of potential antiangiogenic compounds with microvascular (e.g. BACE cells) as opposed to

**Figure 7** Histology of sponge implants. **a**, Day 8 control sponge. **b**, Day 8 after daily injection of bFGF 100 ng into the sponge. **c**, Day 8 after daily injection of bFGF 100 ng and suramin 3 mg into the sponge. **d**, Day 8 after daily injection of bFGF 100 ng and CPD14 1 mg into the sponge.

**Figure 8** Weight loss data for KHT tumour-bearing mice. Control (○), suramin treated (●) and CPD14 treated (□). Suramin (60 mg $kg^{-1}$ i.p.) and CPD14 (42 mg $kg^{-1}$ i.p.) were given on days 1, 5, 9, 13 and 17 after implant. The control group comprised 34 mice. The suramin and CPD14 groups contained eight mice each. Mice received a subcutaneous implant of KHT tumour cells on the back at day 0.

large-vessel (HUVEC) endothelium. Previous studies with PPS only employed large-vessel endothelium, and so we compared the inhibitory activity of suramin, CPD11 and PPS on bFGF-stimulated large-vessel (HUVEC) and capillary (BACE) endothelium. It is clear that while suramin, CPD11 and PPS can block [$^3$H]methylthymidine uptake by HUVECs in response to 1 ng $ml^{-1}$ bFGF, significant inhibition is seen only at millimolar concentrations of inhibitor. In contrast, BACE cells were substantially more sensitive to inhibition by all three inhibitors. Thus $IC_{50}$ values for suramin and CPD11 are in the region of 10 μM, that is 10- to 100-fold lower than for HUVECs. Figure 5c shows that PPS inhibits bFGF-driven HUVEC and BACE cell [$^3$H]methylthymidine uptake, and that BACE cells are inhibited at lower concentrations than are HUVECs. However, in contrast to studies on blocking of conditioned media from SW13 cells transfected with K-FGF, we found that PPS was no more active in blocking bFGF-driven [$^3$H]methylthymidine uptake in either HUVEC or BACE cells than was suramin or CPD11. This may reflect different interactions with members of the FGF family or a difference in experimental conditions, e.g. bFGF concentrations in our assays. Another study (Zugmaier *et al.*, 1992) has shown that PPS given by daily intraperitoneal injection is able to block the growth of tumours in xenografted nude mice in a dose-dependent fashion. However, PPS failed to affect the growth of established tumours (i.e. where the tumour burden exceeded 10 $mm^2$).

CPD14 and CPD15 together with CPD7 form a structural series the members of which differ only in the number of intervening rings between the substituted naphthyl rings (Figure 2). As such they clearly illustrate the absolute requirement of the intervening rings for inhibitory activity. Thus, CPD7 is inactive on BACE cells, whereas CPD14 and CPD15 strongly inhibited both cell types. Interestingly, the most favourable ratio of $IC_{50}$ to MTD was seen with compounds that have two bridging rings, e.g. CPD12 and CPD14 (Table II). This is largely because these compounds have essentially equipotent growth-inhibiting activity to compounds with four bridging rings, but reduced toxicity. In contrast to the clear requirement for bridging rings in the structure, our studies have so far failed to reveal clear structure–activity relationships pertaining to the substitution pattern of the naphthyl rings.

Hori *et al.* (1991) transfected the bFGF gene with an IgG secretion signal added into non-tumorigenic A31 cells and conferred tumorigenicity. Tumorigenicity was strongly antagonised by i.v. administration of anti-bFGF monoclonal antibodies, pointing to a potentially crucial role for bFGF in tumour growth. Similar effects have recently been reported with antibodies that block the activity of VEGF (Kim *et al.*, 1993). The strong interaction of suramin with heparin-binding growth factors (Middaugh *et al.*, 1992) suggests that the analogues may also be effective against other heparin-binding growth factors. It is notable that all of the best-characterised angiogenic factors are also heparin binding (e.g. aFGF, VEGF, placental growth factor, pleiotrophin). Nevertheless, the antiangiogenic activity of suramin-like molecules is unlikely to be attributable solely to attenuation of the interaction of bFGF with its receptor but also to arise from its activities on cells' proliferative apparatus as outlined in the introduction.

We conclude that polyanions of similar structure to the suramin molecule are able to block bFGF-stimulated growth of capillary endothelium *in vitro* and bFGF-driven angiogenesis *in vivo*. These, together with their lower toxicity, offer the opportunity of widening the suramin 'therapeutic window'. CPD12 and CPD14 appear to offer particular promise and are being examined further.

The authors thank Dr Colin Potter, Nuffield Department of Medicine, for access to an automated 96 well harvester and flat-bed beta-plate scintillation counter and John Bowler and Debbie Pocock (MRC RBU) for skilled and dedicated technical assistance with the tumour growth and mouse body weight experiments. T.-P.D. Fan thanks The Wellcome Trust for support.

**Abbreviations:** BACE cells, bovine adrenal capillary endothelial cells; HUVECs, human umbilical vein endothelial cells; FCS, fetal calf serum; bFGF, basic fibroblast growth factor; aFGF, acidic fibroblast growth factor; PDGF, platelet-derived growth factor; IGF-1, insulin-like growth factor 1; PPS, pentosan polysulphate; DMEM, Dulbecco's modified Eagle medium; PBS, phosphate-buffered saline; TLC, thin-layer chromatography; i.p., intraperitoneal.

### References

ANDRADE, S.P., FAN, T.-P.D. & LEWIS, G.P. (1987). Quantitative *in vivo* studies on angiogenesis in a rat sponge model. *Br. J. Exp. Pathol.*, **68**, 755–766.

BAGHDIGUIAN, S., NICKEL, P., MARVALDI, J. & FANTINI, J. (1990). A suramin derivative induces enterocyte-like differentiation of tumour colon cancer cells without lysosomal storage disorder. *Anti-Cancer Drugs*, **1**, 59–66.

BALABAN, I.E. & KING, H. (1927). Trypanocidal action and chemical constitution. VII. s-Carbamides and arylamides of napthylamine-di- and tri-sulphonic acids with some observations on the mesomorphic state. *J. Chem. Soc.*, 3068–3097.

BICKNELL, R. & HARRIS, A.L. (1992). Anti-cancer strategies involving the vasculature. Vascular targeting and the inhibition of angiogenesis. *Sem. Cancer Biol.*, **3**, 399–407.

BOJANOWSKI, K., LELIEVRE, S., MARKOVITS, J., COUPRIE, J., JACQUEMIN-SABLON, A. & LARSEN, A.K. (1992). Suramin is an inhibitor of DNA topoisomerase II *in vitro* and in Chinese hamster fibrosarcoma cells. *Proc. Natl Acad. Sci. USA*, **89**, 3025–3029.

BUTLER, S.J., KELLY, E.C., MCKENZIE, F.R., GUILD, S.B., WAKELAM, M.J. & MILLIGAN, G. (1988). Differential effects of suramin on the coupling of receptors to individual species of pertussis toxin-sensitive guanine-nucleotide-binding proteins. *Biochem. J.*, **251**, 201–205.

COFFEY, R.J., LEOF, E.B., SHIPLEY, G.D. & MOSES, H.L. (1987). Suramin inhibition of growth factor receptor binding and mitogenicity in AKR-2B cells. *J. Cell Physiol.*, **132**, 143–148.

D'AMORE, P.A. & THOMPSON, R.W. (1987). Mechanisms of angiogenesis. *Annu. Rev. Physiol.*, **49**, 453–464.

DENEKAMP, J. & HOBSON, B. (1982). Endothelial cell proliferation in experimental tumours. *Br. J. Cancer*, **46**, 711–720.

FAN, T.-P.D., HU, D.-E. & HILEY, C.R. (1992). Development and validation of a sponge model for quantitative studies on angiogenesis. In *Angiogenesis in Health and Diseases*, M.E. Maragoudakis, P. Gullino & P. I. Lelkes (eds), Plenum Press: New York.

FAWCETT, J., HARRIS, A.L. & BICKNELL, R. (1991). Isolation and properties in culture of human adrenal capillary endothelial cells. *Biochem. Biophys. Res. Commun.*, **174**, 903–908.

FOURNEAU, E., TREFOUEL, J. & VALLEE, J. (1924). Recherches de chimiotherapie dans la serie du 205 Bayer. Urees des acidesaminobenzoyl aminonaphtaleniques. *Annales de L'Institut Pasteur*, **38**, 81.

HENSEY, C.E., BOSECOBOINIK, D. & AZZI, A. (1989). Suramin, anti-cancer drug, inhibits protein kinase C and induces differentiation in neuroblastoma cell clone NB2A. *FEBS Lett.* **258**, 156–158.

HORI, A., SASADA, R., MATSUTANI, E., NAITO, K., SAKURA, Y., FUJITA, T. & KOZAI, Y. (1991). Suppression of solid tumour growth by immortalizing monoclonal antibody against human basic fibroblast growth factor. *Cancer Res.*, **51**, 6180–6184.

HOSANG, M. (1985). Suramin binds to platelet derived growth factor and inhibits its biological activity. *J. Cell Biochem.*, **29**, 265–273.

JAFFE, E.A., NACHMAN, R.L., BECKER, C.G. & MINICK, R.C. (1973). Culture of human endothelial cells derived from umbilical veins. Identification by morphologic and immunologic criteria. *J. Clin. Invest.*, **52**, 2745–2756.

JENTSCH, K.D., HUNSMANN, G., HARTMANN, H. & NICKEL, P. (1987). Inhibition of human immunodeficiency virus type I reverse transcriptase by suramin-related compounds. *J. Gen. Virol.*, **68**, 2183–2192.

KIM, K.J., LI, B., WINER, J., ARMANINI, M., GILLETT, N., PHILLIPS, H.S. & FERRARA, N. (1993). Inhibition of vascular endothelial growth factor-induced angiogenesis suppresses tumour growth *in vivo*. *Nature*, **362**, 841–844.

KOPP, R. & PFEIFFER, A. (1990). Suramin alters phosphoinositide synthesis and inhibits growth factor receptor binding in HT-29 cells. *Cancer Res.*, **50**, 6490–6496.

LATINEN, L. (1987). *Griffonia simplicifolia* lectins bind specifically to endothelial cells and some epithelial cells in mouse tissues. *Histochem. J.*, **19**, 225–234.

LA ROCCA, R.V., STEIN, C.A. & MYERS, C.E. (1990*a*). Suramin: prototype of a new generation of antitumour compounds. *Cancer Cells*, **2**, 106–115.

LA ROCCA, R.V., STEIN, C.A., DANESI, R., JAMIS-DOW, C.A., WEISS, G.H. & MYERS, C.E. (1990*b*). Suramin in adrenal cancer: modulation of steroid hormone production, cytotoxicity *in vitro* and clinical antitumour effect. *J. Clin. Endocrinol. Metab.*, **71**, 497–504.

LA ROCCA, R.V., MYERS, C.E., STEIN, C.A., COOPER, M.R. & UHRICH, M. (1990*c*). Effect of suramin in patients with refractory nodular lymphomas requiring systemic therapy. *Proc. Am. Soc. Clin. Oncol.*, **9**, 268.

MCCARTHY, S.A. & BICKNELL, R. (1992). Responses of pertussis toxin-treated microvascular endothelial cells to transforming growth factor $\beta$1. No evidence for pertussis-sensitive G protein involvement in TGF-$\beta$ signal transduction. *J. Biol. Chem.*, **267**, 21617–21622.

MCCARTHY, S.A., KUZU, I., GATTER, K.C. & BICKNELL, R. (1991). Heterogeneity of the endothelial cell and its role in organ preference of tumour metastasis. *Trends Pharmacol. Sci.*, **12**, 462–467.

MIDDAUGH, C.R., MACH, H., BURKE, C.J., VOLKIN, D.B., DABORA, J.M., TSAI, P.K., BRUNER, M.W., RYAN, J.A. & MARFIA, K.E. (1992). Nature of the interaction of growth factors with suramin. *Biochemistry*, **31**, 9016–9024.

MOSCATALLI, D. & QUARTO, N. (1989). Transformation of NIH3T3 cells with basic fibroblast growth factor or the hst/K-*fgf* oncogene causes downregulation of the fibroblast growth factor receptor: reversal of morphological transformation and restoration of receptor number by suramin. *J. Cell Biol.*, **109**, 2519–2527.

MYERS, C.E., LA ROCCA, R.V., STEIN, C.A., COOPER, M., DAWSON, N., CHOYKE, P., LINEHAM, M. & UHRICH, M. (1990). Treatment of hormonally refractory prostate cancer with suramin. *Proc. Am. Soc. Clin. Oncol.*, **9**, 133.

NAKAJIMA, M., DE CHAVIGNY, A., JOHNSON, C.E., HAMADA, J.I., STEIN, C.A. & NICOLSON, G.L. (1991). Suramin. A potent inhibitor of melanoma heparanase and invasion. *J. Biol. Chem.*, **266**, 9661–9666.

OLANDER, J.V., CONNOLLY, D.T. & DELARCO, J.E. (1991). Specific binding of vascular permeability factor to endothelial cells. *Biochem. Biophys. Res. Commun.*, **175**, 68–76.

PESENTI, E., SOLA, F., MONGELLI, N., GRANDI, M. & SPREAFICO, F. (1992). Suramin prevents neovascularisation and tumour growth through blocking of basic fibroblast growth factor activity. *Br. J. Cancer*, **66**, 367–372.

POLLACK, M. & RICHARD, M. (1990). Suramin blockade of insulin-like growth factor I-stimulated proliferation of human osteosarcoma cells. *J. Natl Cancer Inst.*, **82**, 1349–1352.

SPIGELMAN, Z., DOWERS, A., KENNEDY, S., DISORBO, D., O'BRIEN, M., BARR, R. & MCCAFFREY, R. (1987). Anti-proliferative effects of suramin on lymphoid cells. *Cancer Res.*, **47**, 4694–4698.

WALZ, T.M., ABDIU, A., WINGREN, S., SMEDS, S., LARSSON, S.-E. & WASTESON, A. (1991). Suramin inhibits growth of human osteosarcoma xenografts in nude mice. *Cancer Res.*, **51**, 3585–3589.

WELLSTEIN, A., ZUGMAIER, G., CALIFANO, J.A., KERN, F., PAIK, S. & LIPPMAN, M.E. (1991). Tumour growth dependent on Kaposi's sarcoma-derived fibroblast growth factor inhibited by pentosan polysulfate. *J. Natl Cancer Inst.*, **83**, 716–720.

WILLIAMS, L., TREMBLE, P.M., LEVIN, M.F. & SUNDAY, M.E. (1984). Platelet-derived growth factor receptors form a high affinity state in membrane preparations. *J. Biol. Chem.*, **259**, 5287–5294.

ZUGMAIER, G., LIPPMAN, M.E. & WELLSTEIN, A. (1992). Inhibition by pentosan polysulfate of heparin-binding growth factors released from tumour cells and blockage by PPS of tumor growth in animals. *J. Natl Cancer Inst.*, **84**, 1716–1724.